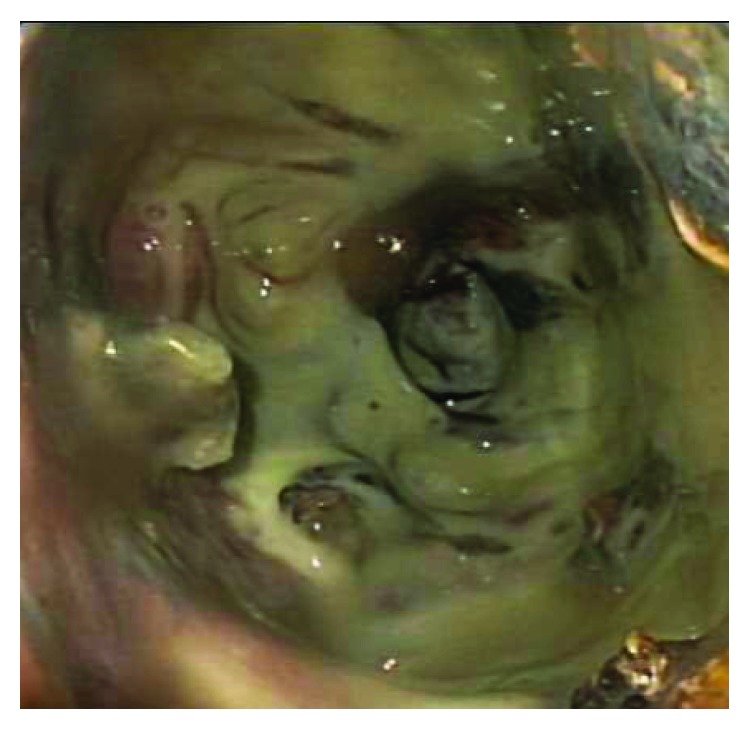# Mechanisms and Management of Acute Pancreatitis

**DOI:** 10.1155/2018/6218798

**Published:** 2018-03-15

**Authors:** Ari Garber, Catherine Frakes, Zubin Arora, Prabhleen Chahal

**Affiliations:** ^1^Department of Gastroenterology and Hepatology, Digestive Disease Institute, Cleveland Clinic Foundation, Cleveland, OH, USA; ^2^Department of Internal Medicine, Medicine Institute, Cleveland Clinic Foundation, Cleveland, OH, USA

## Abstract

Acute pancreatitis represents a disorder characterized by acute necroinflammatory changes of the pancreas and is histologically characterized by acinar cell destruction. Diagnosed clinically with the Revised Atlanta Criteria, and with alcohol and cholelithiasis/choledocholithiasis as the two most prominent antecedents, acute pancreatitis ranks first amongst gastrointestinal diagnoses requiring admission and 21st amongst all diagnoses requiring hospitalization with estimated costs approximating 2.6 billion dollars annually. Complications arising from acute pancreatitis follow a progression from pancreatic/peripancreatic fluid collections to pseudocysts and from pancreatic/peripancreatic necrosis to walled-off necrosis that typically occur over the course of a 4-week interval. Treatment relies heavily on fluid resuscitation and nutrition with advanced endoscopic techniques and cholecystectomy utilized in the setting of gallstone pancreatitis. When necessity dictates a drainage procedure (persistent abdominal pain, gastric or duodenal outlet obstruction, biliary obstruction, and infection), an endoscopic ultrasound with advanced endoscopic techniques and technology rather than surgical intervention is increasingly being utilized to manage symptomatic pseudocysts and walled-off pancreatic necrosis by performing a cystogastrostomy.

## 1. Introduction

Acute pancreatitis (AP), simply defined, represents a disorder characterized by acute necroinflammatory changes of the pancreas. The purpose of this review is to explore the historical, epidemiologic, histologic, and pathologic mechanisms underpinning the disease and the current evidenced-based management algorithms.

## 2. Historical Perspective

From the Greek roots “pan” (all) and “kreas” (flesh or meat), the term “pancreas” was first coined by Ruphos of Ephesus (c. 100 CE), to describe an organ that had no cartilage or bone. Despite its early roots, it was not until much later that the first clinical description of acute pancreatitis by Nicholaes Tulp (1593–1674), a Dutch anatomist, was published [1]. However, amidst much speculation of causality, the first systematic assessment of acute pancreatitis was authored by Reginald Fitz (1843–1913) in his entitled review “Acute Pancreatitis: A Consideration of Hemorrhage, Hemorrhagic, Suppurative, and Gangrenous Pancreatitis, and of Disseminated Fat Necrosis,” highlighting alcohol, gallstones, and other etiologic factors. Claude Bernard (1813–1878) is credited as one of the early pioneers of pancreatic physiology, identifying pancreatic juice's capability of converting starch into sugar and emulsifying lipids into their constituents. Further classification, prognostication, and understanding of the pathogenic mechanisms have led to the burgeoning field of pancreatology, and the management of this complex pancreatic disease is the subject of this review.

## 3. Epidemiology

Acute pancreatitis is the number one gastrointestinal diagnosis prompting inpatient admission and ranks 21st on the list of all diagnoses requiring hospitalization. The incidence of acute pancreatitis ranges from 13 to 45/100,000 with equal affinity for each gender (though with differing etiologies) [2]. Acute pancreatitis secondary to alcohol is more common in men, whereas gallstone pancreatitis is more common in women and appears to affect African Americans disproportionately for unclear reasons. In 2009, the Healthcare Cost and Utilization Project National Inpatient Sample identified 274,119 individuals discharged from the hospital with acute pancreatitis, representing a 30% increase from 2000 and necessitating a median length of stay of 4 days. Acute pancreatitis contributed to, and/or was responsible for, 8653 deaths in 2009, representing an underlying cause of death rate of 1 per 100,000 and ranking it as the 14th leading cause of gastrointestinal death with a cost of 2.6 billion dollars in inpatient expenses [3].

## 4. Embryology, Anatomy, Histology

Embryologically, the pancreas is an endodermal structure that is the product of the fusion of the ventral and dorsal pancreas at approximately 8 weeks' gestation. The celiac artery (via the superior pancreaticoduodenal artery) and the superior mesenteric artery (via the inferior pancreaticoduodenal artery) provide the arterial blood supply to the pancreas. Venous drainage of the pancreas occurs through the splenic and superior mesenteric veins, which drain into the portal vein.

The functional pancreas itself is divided into endocrine and exocrine components. The exocrine pancreas (comprised of acinar cells and ductal tissue) represents approximately 85% of pancreatic tissue and is responsible for zymogen and bicarbonate secretion into the duodenum [4]. The endocrine pancreas (comprised of the islets of Langerhans, itself comprised of alpha, beta, and delta cells) is responsible for hormonal secretion (glucagon, insulin, and somatostatin, resp.) into the general circulation.

Acinar cell destruction is the histologic hallmark of acute pancreatitis, a consequence of autodigestion secondary to zymogen activation. It is believed that premature activation of trypsin is the inciting event leading to the inflammatory cascade culminating in acute pancreatitis [5].

Histologically, three patterns of acute pancreatitis have been recognized. Type 1 necrosis (the predominant histologic form) refers to necrosis principally affecting perilobular, interlobular, or peripancreatic fatty tissue. Type 2 necrosis shows a predominant ductal involvement of necrosis. Type 3 necrosis involves only the acinar cell itself [6].

## 5. Diagnosis

The Revised Atlanta Criteria of 2012 (updated from 1992) requires two of three conditions be met to diagnose acute pancreatitis: (1) abdominal pain consistent with acute pancreatitis (i.e., epigastric abdominal pain with possible radiation to the back), (2) lipase or amylase ≥ 3 times the upper limit of normal, and or (3) characteristic imaging features of acute pancreatitis as noted on CT, MRI, or ultrasound [7]. However, imaging of the pancreas is recommended only in patients whom the diagnosis is unclear, for those who fail to improve within the first 48–72 hours, or to assess for complications (described below) [8]. Onset (time zero) refers to the timing of when abdominal pain began, not hospital admission.

**Figure 1:**
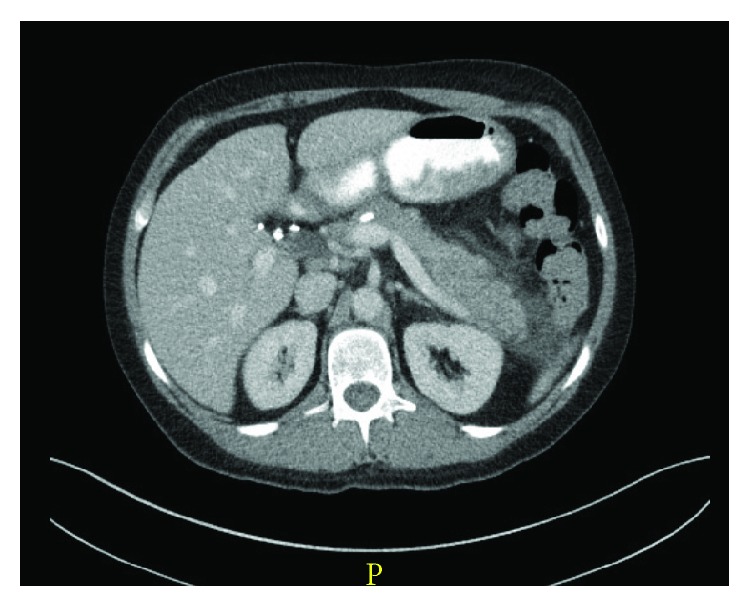


**Figure 2:**
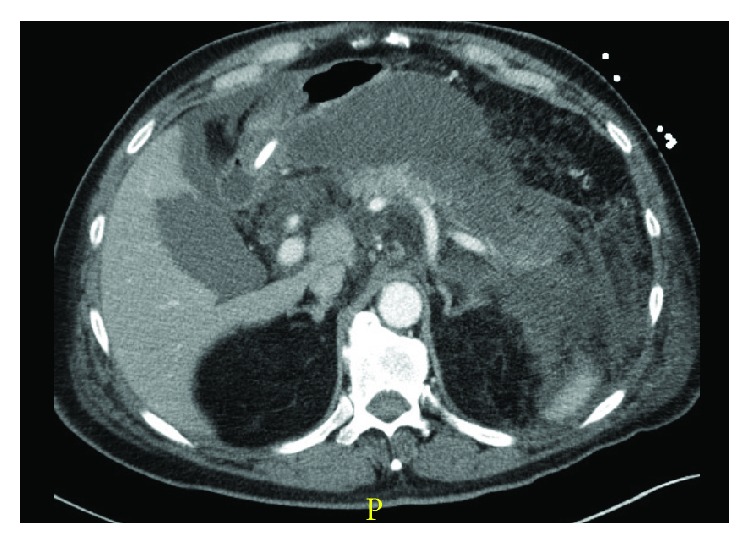


Acute pancreatitis is further classified into two separate categories: interstitial edematous (Figure 1, where the pancreas shows evidence of diffuse enlargement and enhancement due to inflammatory edema without evidence of necrosis) and necrotizing (Figure 2, where cell death of the pancreatic and or peripancreatic tissue is observed). The latter occurs in approximately 5–10% of cases of acute pancreatitis [7]. Necrotizing pancreatitis is further subclassified into sterile or infected.

## 6. Etiology

The two most common causes of acute pancreatitis are cholelithiasis/choledocholithiasis and alcohol (definitions vary as does duration with consumption between 50 and 80 grams or 4–7 drinks/day) with frequency estimates of 40% and 30%, respectively. The other etiologies are hypertriglyceridemia (typically >1000 mg/dL), medications, trauma, infections, iatrogenesis (surgical or post-ERCP), genes, anatomy (pancreatic divisum, sphincter of Oddi dysfunction which remain controversial), and autoimmunity [9]. What follows is a discussion of a few of these important etiologies.

Medications thought to induce acute pancreatitis have been classified on the level of evidence to support the association. Class I medications are defined as those where recurrence of acute pancreatitis was confirmed upon rechallenging. Class I is further subdivided into 1a (other causes of pancreatitis ruled out) and 1b (alternative etiologies not ruled out). Class II medications do not meet strict criteria for class 1 but exhibit a consistent latency period in a preponderance of reported cases. Class III and IV medications refer to those in which two or one published case report of medication-induced pancreatitis has been reported, respectively. Some common Class 1a and 1b medications include amiodarone, all-*trans*-retinoic acid (ATRA), 6-MP/azathioprine, dexamethasone, enalapril, furosemide, hydrocortisone, isoniazid, losartan, mesalamine, metronidazole, methyldopa, omeprazole, pravastatin, simvastatin, trimethoprim-sulfamethoxazole, tetracycline, and valproic acid [10].

Mutations, upregulation, and genetic variants in several genes have been implicated in acute pancreatitis, namely, the trypsinogen gene (PRSS1) and trypsin inhibitor (SPINK1), cystic fibrosis transmembrane regulator (CFTR) variants, and endothelial ion/water channel CLDN2 risk allele [5].

The exact pathologic mechanism by which gallstones cause pancreatitis remains unclear though it is hypothesized that either choledocholiths (typically stones < 5 mm) impinge on the adjacent pancreatic duct or lodge in the ampulla causing increased pressure, reflux of pancreaticobiliary secretions, and acinar cell secretion into the interstitium leading to the inflammatory cascade [11].

Post-ERCP pancreatitis estimates range from 1.6% to 15.7%, and a meta-analysis of 21 studies found an incidence of approximately 3.5% [12, 13]. Freeman identified young age, biliary sphincter balloon dilation in intact papilla, pancreatic duct contrast injection, normal bilirubin, precut sphincterotomy or pancreatic sphincterotomy, and suspected sphincter of Oddi dysfunction as risk factors for post-ERCP pancreatitis [14].

Autoimmune pancreatitis is a predominantly lymphocytic inflammatory process that results in eventual organ fibrosis and dysfunction. While many diagnostic criteria have been promulgated, the modified Japan Pancreas Society Criteria require a combination of typical imaging (CT, MRCP, or ERCP) and either serology (IgG4 or IgG totals, etc.) or pancreaticobiliary/extraintestinal findings (sialadenitis, nephritis, or IgG4 pneumonitis) for diagnosis [15].

## 7. Complications

Two known local complications of pancreatitis are pseudocysts and walled-off necrosis. Both are walled-off encapsulated collections that usually mature 4 weeks after the initial acute pancreatitis episode. The difference lies in the fact that a pseudocyst has a homogenous fluid density whereas walled-off necrosis describes both fluid and nonfluid heterogeneous components which represent necrotic debris with or without loculation [7]. Pseudocysts are the product of pancreatic/peripancreatic fluid collections, whereas walled-off necrosis is the product of initial pancreatic/peripancreatic necrosis.

Organ failure in acute pancreatitis is defined by the modified Marshall score, which assesses the degree of dysfunction in three organ systems (cardiovascular, renal, and pulmonary). Each organ system is scored on a scale of 0–4, and any organ that is scored as 2 or above meets criteria for organ failure. The cardiovascular, pulmonary, and renal systems receive a score greater than 2 when the following are identified (resp.): systolic blood pressure is <90 mmHg and not responsive to fluids, PaO_2_/FiO_2_ ratio of 201–300, and serum creatinine 1.9–3.6 mg/dL. [16].

## 8. Severity

Mortality in the setting of acute pancreatitis has been estimated at 5%, though when stratified into interstitial versus necrotizing (3% versus 17%), and in the necrotizing subset, infected versus sterile (30% versus 12%), the range is quite variable [17]. Several models have been promulgated to predict the initial severity of the acute pancreatitis episode with the most common indices described below.

The well-recognized Ranson criteria is one of the earliest predictive models, but is difficult to utilize in clinical practice. It requires 5 parameters on admission and 6 parameters after 48 hours of hospitalization. A meta-analysis of 110 studies showed that it was a poor predictor of severity, with a high false positive rate [18].

The Acute Physiology and Chronic Health Examination (APACHE) II score comprises 12 physiologic measures (Glasgow coma scale, leukocyte count, hematocrit, creatinine, potassium, sodium, pH/HCO_3_, respiratory rate, arterial-alveolar gradient, heart rate, mean arterial pressure, and temperature) and extra points for age and chronic diagnoses. Scores less than 8 on admission and at 72 hours portend a mortality less than 4% with risk increasing to 11–18% with scores > 8 [17].

The systemic inflammatory response syndrome (SIRS criteria), a scoring system that assigns a point to the presence of various thresholds in temperature, respiratory rate, leukocyte count, and heart rate (and considered present when two or more criteria are met), has been used to predict pancreatitis severity, with SIRS presence on the day of admission indicating increased risk of severe disease [19].

The bedside index of severity in acute pancreatitis (BISAP) score uses a one point scoring system with each component of the indexes: BUN > 25 mg/dL, altered mental status, SIRS (as described above), age > 60, and the presence of pleural effusions with mortality ranging from <1% (BISAP = 0) to 22% (BISAP = 5) [20].

## 9. Treatment

The management of acute pancreatitis depends on the severity of disease and the concomitant complications that may arise. Our discussion begins with uncomplicated disease and then expands to more complex clinical scenarios.

### 9.1. Fluid Resuscitation

The disease process leads to acinar cell injury and the consequent proinflammatory cytokine cascade leads to microvascular permeability, interstitial edema, vasoconstriction, and eventual decreased capillary perfusion in animal models. Pancreatitis also causes hypovolemia by inducing poor oral intake, insensible losses, third-spacing of fluids, and emesis. Therefore, fluid resuscitation has become the cornerstone of conservative treatment [21]. In the absence of cardiac, pulmonary, or renal contraindications, various recommendations on the initial fluid resuscitation regimen have varied from 250–500 cc/hr with or without bolus to achieve hemodynamic stability, targeting a mean arterial pressure > 60 or simply targeting a urine output > 0.5 cc/kg/hr [22–25]. While no specific targets are currently recommended, hemodilution (decreased hematocrit), reduced uremia (indicating adequate kidney perfusion), and normalization or maintenance of normal creatinine have been proposed. A practical, evidence-based approach to fluid resuscitation is needed [26–28].

With respect to timing, early resuscitation has been shown to decrease the risk of SIRS, ICU admission, organ failure, and length of stay. Even though the exact duration of aggressive hydration remains unclear, the first 24 hours appear to be paramount [29, 30]. In addition, the type of fluid may make a difference as well. In a randomized controlled study performed by Wu and colleagues comparing the effectiveness of normal saline and lactated ringers in acute pancreatitis, the authors found a significant reduction in SIRS and CRP levels in those who received lactated ringers [28]. These findings, in conjunction with possible nonanion gap metabolic acidosis with normal saline make lactated Ringer's solution preferable. Thus, we utilize a total infusion of 2500–4000 mL in the first 24 hours while reassessing noninvasive clinical targets and biochemical targets every 6–8 hours.

### 9.2. Nutrition

Current data supports early resumption of a low-fat solid diet with mild acute pancreatitis. While it does not lead to a shorter length of hospital stay or decreased 30-day readmission rate, a randomized trial evaluating the tolerance of a low fat solid meal versus a liquid diet showed no increased adverse events (pain/nausea necessitating cessation) and led to increased caloric intake [31]. Moreover, it appears that it is safe to initiate oral intake in mild acute pancreatitis on admission and that one does not have to wait for the pancreas to “cool down” per se [32]. Randomized controlled trial data of enteral versus parenteral nutrition in severe pancreatitis has shown a decreased incidence of pancreatic infectious complications such as infected necrosis, abscess, and multiorgan failure [32]. Enteral nutrition prevents bacterial translocation by maintaining the intestinal barrier. The benefit of initiating enteral nutrition does not appear to extend beyond 48 hours of admission, as no reduction in mortality, infectious complications, or multiorgan failure was recognized when initiated beyond that point [33].

The benefit of nasogastric versus nasojejunal feeds was evaluated in a randomized trial of 78 patients which showed that nasogastric feeding was not inferior to nasojejunal feeding with no difference in secondary endpoints such as pain, intestinal permeability (measured by lactulose/mannitol excretion), and endotoxemia (as measured by immunoglobulin core G and M endotoxins) [34]. Thus, we utilize nasojejunal feeds in those unable to tolerate oral feeding.

### 9.3. Role of Endoscopic Retrograde Cholangiopancreatography (ERCP)

The role of ERCP in patients with AP is generally reserved for acute biliary pancreatitis secondary to choledocholithiasis. While many scoring systems and algorithms have been developed, the proposed strategy to assign risk of choledocholithiasis proposed by the American Society for Gastrointestinal Endoscopy is the most widely used. It stratifies predictors of choledocholithiasis into very strong (observed on US, cholangitis or total bilirubin > 4 mg/dL), strong (CBD > 6 mm with gallbladder in situ or total bilirubin between 1.8 and 4 mg/dL), and moderate (abnormal AST/ALT or alkaline phosphatase, clinical gallstone pancreatitis, or age > 55). When a patient has one very strong predictor or two strong predictors, the risk of choledocholithiasis is high. All other predictors are considered intermediate and no qualifying predictors is considered low risk [35, 36].

In patients with mild biliary pancreatitis with improving signs and symptoms, ERCP preceding cholecystectomy has limited value and may be harmful. In these cases, magnetic resonance cholangiopancreatography (MRCP) or endoscopic ultrasound (EUS) can be used for diagnostic purposes [8].

Rectally administered indomethacin has been shown in a multicenter, randomized, placebo-controlled, double-blind clinical trial of 602 high risk patients to reduce the risk of postprocedural pancreatitis and the severity of pancreatitis in those who subsequently developed symptoms [37]. However, in a similar study of 449 predominantly average risk patients undergoing ERCP, no clinical benefit was observed and the study was stopped due to futility [38]. Thus, rectally administered indomethacin could be considered in high risk patients prior to ERCP, as it is easy to utilize, inexpensive, and safe.

### 9.4. Antibiotics

Antibiotic prophylaxis in the absence of suspected or confirmed infection is not recommended. Apart from imipenem, no decrease in pancreatic infection risk or mortality has been observed with prophylactic antibiotic use [39]. Further randomized trials utilizing prophylactic antibiotics have failed to show benefit [8]. In the setting of confirmed or suspected pancreatic infection (infected pseudocyst or necrosis), prompt use of regimens known to penetrate pancreatic necrosis are recommended (quinolones and metronidazole, or carbapenems).

### 9.5. Cholecystectomy

Cholecystectomy should be performed on initial hospitalization in patients with acute biliary pancreatitis. Systematic review of 9 studies involving 998 patients with mild biliary pancreatitis showed that early cholecystectomy in the setting of gallstone pancreatitis (i.e., during the index admission) reduced the incidence of recurrent admissions for repeat biliary-related events including pancreatitis, cholecystitis, and biliary colic. Early cholecystectomy was not associated with increased adverse events including mortality nor conversion from a laparoscopic procedure to an open procedure [40].

### 9.6. Management of Persistent Fluid Collections or Infected Necrosis

**Figure 3:**
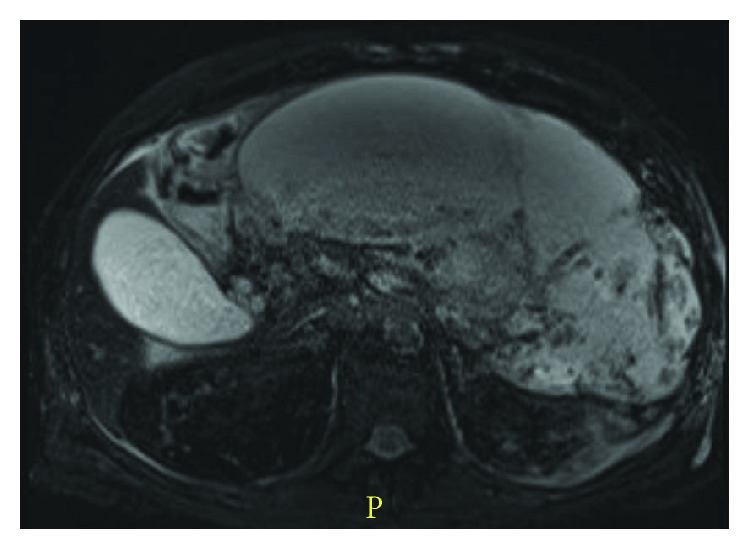


**Figure 4:**
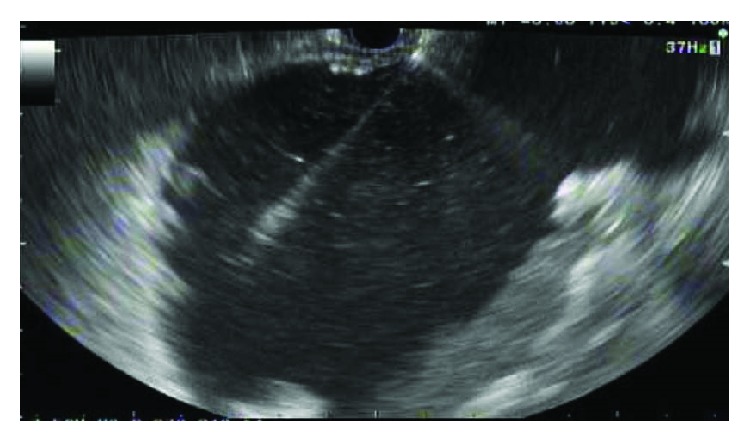


We intervene upon pancreatic fluid collection or infected necrosis only when there are significant symptoms present, including persistent abdominal pain, gastric outlet obstruction, fluid leakage due to disconnected pancreatic duct, and infection [41]. It is crucial to classify fluid collections as either pseudocyst or walled-off pancreatic necrosis because of the differences in prognosis and treatment. CT imaging can underestimate the existence of necrotic debris; therefore, MRI (Figure 3) and endoscopic ultrasound (EUS) (Figure 4) are better for assessment [41]. The management has changed from what historically was a surgical intervention to now less invasive approaches. The approach to managing these complications is discussed below.

### 9.7. Open Surgical Drainage

Open necrosectomy is performed via laparotomy through a subcostal incision, where blunt removal of all necrotic tissue is done [42]. Early conservative management with late surgical intervention is superior to early necrosectomy [43]. Surgery is delayed preferably four weeks after onset of disease, as this is thought to allow for time for the acute necrotic collection to mature and demarcate, hereby facilitating necrosectomy [44]. In a recent randomized control trial, open necrosectomy had a high rate of complications or death (69%) [43]. Those undergoing open necrosectomy also had a higher rate of long-term complications, including incisional hernias (24%), new onset diabetes (38%), and use of pancreatic enzymes (33%). Therefore, therapy has shifted toward a minimally invasive “step-up” approach. This approach starts with more conservative techniques (percutaneous, laparoscopic, and endoscopic) first and then reserving surgery for cases of salvage therapy [43].

### 9.8. Minimally Invasive Techniques

There are several different types of noninvasive techniques to drain and debride persistent fluid collections or infected pancreatic necrosis, including image-guided percutaneous drainage, laparoscopy, and retroperitoneoscopy [45].

Using ultrasound or CT guidance, percutaneous drain placement allows for external access to the area of necrosis to be obtained [46]. A considerable number of patients can be treated with percutaneous drain (PCD) alone without the need for surgical necrosectomy [47]. The PANTER trial found that 35% of their patient population undergoing drainage did not need further surgery [43]. A systematic review by van Baal et al. showed that percutaneous drainage alone was successful in 56% of cases [47]. In the patients who did need surgery, drain placement delayed operative management for several weeks, by allowing for sepsis control [48]. Complications of percutaneous drain placement are pancreaticocutaneous and pancreaticoenteric fistulas (most common), as well as procedure-related complications (i.e., bleeding, colonic perforation, abdominal pain, pneumothorax, or catheter dislodgment) [47].

Transperitoneal laparoscopy is generally not supported because of the technical difficulty and risk of contamination of the peritoneal cavity [45].

Video-assisted retroperitoneal debridement (VARD) is an endoscopic necrosectomy performed over a dilated percutaneous drain tract. A 5 cm subcostal incision is made in the left flank, the necrosis is initially moved with grasping forceps, and the videoscope is inserted. Residual necrosis is removed with laparoscopic grasping forceps [49]. The PANTER trial assigned patients with pancreatic necrosis to either primary open necrosectomy or a step-up approach, where PCD drain was placed initially followed by minimally invasive retroperitoneal necrosectomy when needed. It showed that a minimally invasive step-up approach was associated with lower rate of major complications and death when compared to open necrosectomy [43].

### 9.9. Endoscopic Techniques in the Management of Persistent Fluid Collections or Infected Necrosis

Over the last two decades, endoscopic ultrasound- (EUS-) guided intervention of PFCs and infected necrosis has significantly evolved. There are multiple techniques for the drainage of PFCs including lumen-apposing metal stents (LAMS), direct endoscopic necrosectomy (DEN), and a double-pigtail plastic stent [41]. The TENSION trial is currently underway and will compare the surgical step-up approach versus an endoscopic step-up approach [50].

**Figure 5:**
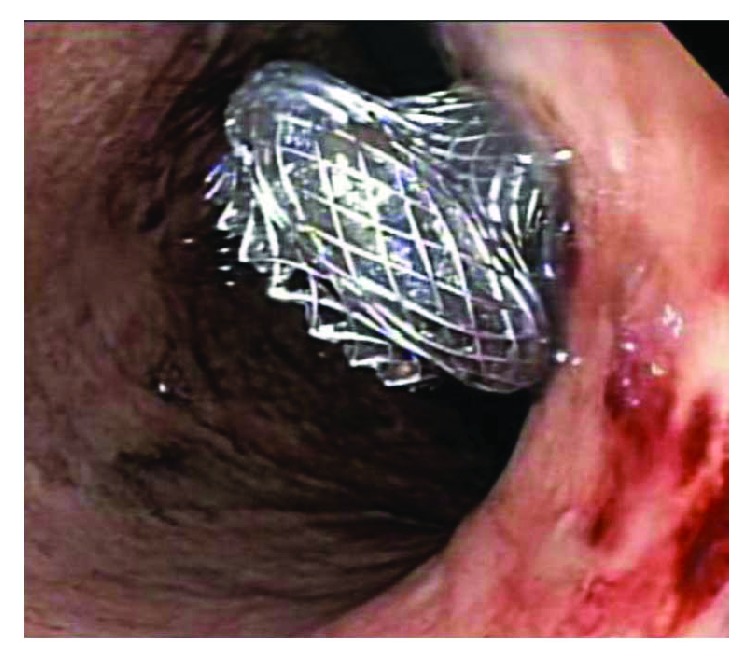


While there are no absolute size guidelines as to when to intervene, encapsulated areas less than 3 cm do not allow placement of a stent for drainage [51]. The necessity for a mature wall around a pseudocyst or walled-off pancreatic necrosis is imperative, as endoscopic cystogastrostomy can lead to free perforation in its absence. It is recommended that the luminal wall and the target cyst or walled-off necrosis lie within 10 mm of the gastrointestinal lumen as evaluated on an endoscopic ultrasound. This ensures technical success and allows the practitioner to assess for pseudoaneurysms and other vascular structures prior to intervention [51, 52]. Pseudocyst contents tend to be fluid and therefore one to two 7–10 Fr pigtail stents are often sufficient for drainage (unless multiple pseudocysts necessitate otherwise). On the other hand, walled-off necrosis often requires multiple stents (given the debris) or a large-caliber fully covered metal stent or lumen-opposing stents such as the Axios™ stent (Figure 5) [53]. Some centers manage WOPN with a hybrid technique involving percutaneous large caliber drain placement for irrigation and endoscopic cystogastrostomy creation working an egress route for irrigation and lavage. Given complications with surgical management approaching 24% with mortality rates reported around 5.8%, minimally invasive endoscopic techniques are considered optimal when expertise is readily available [54].

The technique of direct endoscopic necrosectomy (DEN) involves utilization of an endoscopic ultrasound to visualize the fluid collection with the subsequent fistulous tract made large enough to allow for the passage of the endoscope for debridement and visualization of walled-off pancreatic necrosis (Figure 6). Mechanical cleaning and removal of necrotic debris is then performed [55]. A retrospective analysis has shown that direct endoscopic debridement is feasible with initial success rates of 80% of patients and long-term clinical efficacy in 68% [56]. This endoscopic procedure was shown in one recent RCT to reduce the proinflammatory response (measured with IL-6 levels) and risk of procedure-related complication, in comparison to surgical necrosectomy [57]. Thus, minimally invasive management of complications of AP is presently the standard of care.

## 10. Conclusion

Acute pancreatitis remains a frequent cause of hospital admission necessitating a multipronged approach for the diagnosis and management. While its antecedents remain multifactorial, as are the number of scoring systems that define severity, treatment is predominantly geared toward supportive care with advanced endoscopic adjuncts (in the setting of choledocholithiasis, symptomatic pseudocysts, or walled-off pancreatic necrosis) and early surgical intervention (i.e., cholecystectomy in the setting of an index admission for gallstone pancreatitis) utilized when clinically indicated.

## Figures and Tables

**Figure 6:**